# Mechanistic interactions between *Plasmodium falciparum* and Epstein-Barr virus in endemic Burkitt Lymphoma pathogenesis: a narrative review (2010–2026)

**DOI:** 10.1186/s13027-026-00780-5

**Published:** 2026-07-15

**Authors:** Natalia A. Msami

**Affiliations:** School of Public Health, University of California, Berkeley, CA USA

**Keywords:** Plasmodium falciparum, Malaria, Epstein-Barr virus, Endemic Burkitt Lymphoma, Viral-parasitic interactions

## Abstract

Endemic Burkitt Lymphoma, characterized by an MYC translocation during B-cell activation, is an aggressive, fast-proliferating cancer that predominantly accounts for more than half of all childhood cancers in sub-Saharan Africa. Although a well-established epidemiologic association exists between Epstein-Barr virus infection and *Plasmodium falciparum* malaria, disentangling their mechanistic interactions in the pathogenesis of endemic Burkitt Lymphoma remains difficult. To address this challenge, a comprehensive narrative review of studies published between 2010 and 2026, was conducted. Evidence from mouse models, in vitro and ex vivo studies was synthesized to identify potential key effector cells, cytokine groups, and molecular pathways likely involved in fostering an immune environment that supports lymphomagenesis. The synthesized evidence informed the development of a speculative multi-hit model of endemic Burkitt Lymphoma, in which chronic *Plasmodium falciparum* exposure reconfigures the immune landscape through sustained inflammation, altered effector cell function, and impaired immune surveillance. Within this framework, these changes may subsequently facilitate Epstein-Barr virus persistence, intermittent B-cell reactivation, and cumulative genomic instability, ultimately increasing the likelihood of MYC translocations and the development of endemic Burkitt Lymphoma. The review also proposes incorporating emerging immune effector populations into this conceptual multi-hit model. Although several of the mechanistic relationships explored remain inferential, the framework identifies potential therapeutic and preventive immune targets that warrant further investigation in experimental and longitudinal human studies in high-burden regions.

## Background

Burkitt Lymphoma (BL) is a highly aggressive subtype of B lymphocyte non-Hodgkin lymphoma (NHL) [1–3], characterized by translocation of the MYC proto-oncogene to an immunoglobulin locus during rapid B-cell proliferation and gene rearrangement following activation [2]. Although its occurrence is global, BL prevalence and incidence cases are disproportionately concentrated among children, with marked disparities in cumulative incidence, treatment outcomes, and survival across high-, middle-, and low-income countries [4, 5]. As of 2021, global burden of disease estimates indicated that the incidence rate of BL was 0.20 per 100,000, representing a 31.45% increase since 1990 [6], with school-aged, predominantly male children most affected [7]. Global mortality trends additionally demonstrated a 24.7% increase in BL-associated deaths between 1990 and 2021 [7], with the highest historical incidence reported in eastern (e.g., Malawi and Uganda) and central (e.g., Cameroon) regions of Africa [8]. BL is classified into three distinct clinical variants – endemic BL (eBL), sporadic BL (sBL), and HIV-associated BL – each with characteristic differences in geographic distribution, epidemiological risk factors, patterns of occurrence, and clinical presentation [1, 3, 9]. Cases of eBL occur predominantly in warm, low-lying, malaria-endemic regions of sub-Saharan Africa (SSA), with additional cases reported in Papua New Guinea, and primarily affect children aged 4–7 years [3, 10]. In contrast, sBL and HIV-associated BL occur worldwide, across all climate zones, and affect individuals of all ages [3, 9, 10].

Clinical manifestations of BL differ by disease variant and site of origin. eBL typically presents with rapidly enlarging jaw or facial tumors, sBL often manifests as abdominal tumors, and HIV-associated BL commonly involves tumors of the lymph nodes, bone marrow, and central nervous system (CNS) [3, 10]. Given the disease’s aggressive nature, treatment across all BL variants requires intensive, high-dose chemotherapy protocols to minimize tumor burden. In high-income countries (HIC), most regimens achieve approximately 90% event-free survival (EFS), defined as the time from treatment to disease recurrence or relapse, and high cure rates, defined as “long-term disease-free” survival without relapse (although “long-term” is not numerically defined in the literature) [4, 11]. In low- and middle-income countries (LMICs), however, lower-dose treatment protocols are prioritized due to resource constraints, resulting in cure rates well below 90% and EFS rates anywhere between 40 and 60% and less than 30% for advanced-stage disease [4, 11]. In addition to lower-dose regimens, management of BL in LMICs is further compromised by factors associated with “misdiagnosis, […] treatment delays, refusal or abandonment of treatment, and lack of financial support,” among many others [12]. In an evaluation of treatment outcomes in Mwanza, Tanzania, reported 1-year EFS was 29.6%, with treatment abandonment observed in 34% of cases [4]. Overall survival (OS) rates across LMICs at approximately two years post-diagnosis range from 30% to 45% [13–15], starkly in contrast to outcomes observed in HICs and underscoring persistent global inequities in cancer care [16].

### Associative cofactors in endemic Burkitt Lymphoma

Among the three BL variants, eBL remains the most prevalent, with the highest incidence and disease burden observed in equatorial Africa, often referred to as the “lymphoma belt” [7]. In this region, eBL accounts for almost half of all respective childhood cancers, with incident rate data from 2018 showing eBL in this region estimated at 0.86 per 100,000 among adolescent males, and 0.42 per 100,000 among adolescent females [17]. The geographic clustering of eBL closely overlaps with regions of intense malaria transmission and high prevalence of Epstein-Barr virus (EBV) infection, implicating both pathogens as key etiologic cofactors [18–20]. EBV, a ubiquitous human herpesvirus that infects nearly 90% of the global population during early childhood, is associated with a range of diseases and fatal malignancies, largely due to its immune-evasion strategies and latency programs [2, 21, 22].

The virus was first identified in 1964 by Dr. Anthony Epstein in tumor samples from children affected with eBL in Kampala, Uganda, marking the first established link between a virus and an aggressive human cancer [23]. Since this discovery, numerous studies have demonstrated a strong association between EBV and eBL, with EBV detected in approximately 76.5% of BL cases in equatorial Africa [24], where it plays a critical role in driving the transformative alterations of B lymphocytes necessary for tumorigenesis.

The widespread occurrence of eBL in malaria-endemic regions further suggests that malaria may contribute to BL development, with many studies associating *Plasmodium falciparum* (Pf), the most virulent species within the genus, with lymphoma incidence [18]. Incidence and infection with the Pf parasite are responsible for more than 90% of the world’s malaria-associated mortality, with SSA bearing a disproportionate share of the total global burden. Of the 244.4 million Pf-related malaria cases reported globally in 2022, for instance, 96% (234.8 million) occurred in SSA [25]. Although preventive mechanisms and mass antimalarial treatment programs have successfully reduced the malaria mortality in the region by an estimated 57% between 1990 and 2015 [26], recent data display a decrease in preventive progress and a resurgence of cases, with 2022 marking the highest incidence in malaria case counts since 2004 [25]. When considering the parasite’s involvement with eBL, population-level studies have identified parasite density, genetic diversity in Pf strains, and repeated cumulative infections, as prominent risk factors for eBL incidence in equatorial Africa [27–30]. Notably, eBL incidence increases in parallel with Pf transmission intensity, with rural populations experiencing the highest disease burden.

Given the significant association between Pf transmission and eBL risk, evaluating the potential role of climate change in future eBL trends requires first examining its effects on Pf transmission. Research investigating weather conditions and their influence on Pf transmission reveals the intricate nature of this relationship, with some studies correlating higher temperatures with higher malaria incidence, while others report weak or inconsistent associations [31]. In a recent statistical modeling study predicting Pf incidence from 2024 to 2050, extreme weather conditions were estimated to contribute an additional 123 million clinical malaria cases in holoendemic regions, suggesting that extreme weather events are likely to intensify malaria transmission in areas where the pathogen is firmly established [32]. Although no direct studies have examined the relationship between climate variability and eBL incidence in SSA, the established association between chronic Pf exposure and eBL, combined with projections of climate-driven increases in malaria transmission, supports a plausible increase in eBL burden in high-risk regions over time. This relationship, however, remains spurious and thus requires substantial epidemiological investigation to accurately determine the direction and magnitude of the climate-mediated effect. Overall, emerging evidence from the current literature emphasizes the urgent need for localized, scaled-up, and climate-informed interventions to control Pf transmission and, consequently, mitigate the downstream effects of eBL in vulnerable communities.

### Immune evasion mechanisms and current gaps in understanding

A defining molecular hallmark of all BL variants is the translocation of the proto-oncogenic transcription factor, MYC, during B lymphocyte activation. Thus, understanding the adaptive immune pathways hijacked by each causative factor that ultimately facilitates this event is vital for identifying novel therapeutic and preventive targets.

EBV’s mechanism of action involves directly targeting and infecting activated B lymphocytes in the oropharynx, the primary site of exposure, and inducing a state of latency within the infected B cells, ensuring persistent infection [22, 23]. Fusion of EBV into a B lymphocyte involves interactions between the virus’s glycoproteins, particularly gp350/220, and the B cell’s corresponding complement receptor type 2 (CR2). Once fused into the cell, the virus enters one of four latency programs, integrating its viral genome into the host nucleus, with each program corresponding to a key stage of B cell activation. To evade immune surveillance, EBV expresses genes and secretes proteins that mimic those secreted by B cells during normal activation processes, whether at the naïve, germinal center (GC), or memory stages [23]. Through these programs, the virus effectively alters immune processes associated with antigen presentation, effector cell engagement, and apoptotic signaling [33]. The incidence of eBL is most strongly associated with EBV-infected B lymphocytes in a latency I program, a program characteristic of resting memory B cells, in which viral antigen expression is highly restricted. This immune-silent state creates a microenvironment that facilitates unchecked proliferation of infected cells and, following mutations, their eventual malignancy, without interaction with appropriate effector cells. [34].

Like EBV, Pf employs sophisticated immune-evasion strategies throughout its life cycle to ensure its survival within the infected host. Following transmission by the female Anopheles mosquito, the Pf lifecycle involves interactions with the liver, where replication and proliferation occur, and subsequent invasion of red blood cells (RBCs), in which surface antigens, namely *P. falciparum* erythrocyte membrane protein 1 (PfEMP1), are expressed to prevent splenic clearance [35]. Corresponding antibodies from activated B lymphocytes dominantly target RBCs expressing PfEMP1 on their surfaces, termed the erythrocytic blood stage, with other antibodies geared at the pre-erythrocytic stage. To evade immune recognition, PfEMP1 exhibits high antigenic variation, a major driver of recurrent specific and polyclonal B-cell activation [36]. As reported in the literature, recurrent exposure and infection with Pf, though an indicator of clinical immunity, typically disrupt B cell homeostasis, resulting in the constant reactivation of naïve and memory B lymphocytes, which ultimately creates high proportions of translational (naïve) and atypical (exhausted-state) memory B cells, with dysregulated and impaired functions [35–37]. The increased presence of these atypical B cell subtypes likely contributes to an immunologic milieu that both sustains parasitemia and increases susceptibility to EBV-driven lymphomagenesis.

Given that both EBV and Pf interfere with B lymphocyte immune responses through their respective evasion strategies, their interaction in the development of eBL warrants further investigation. While extensive epidemiological research has established associations among EBV, Pf, and eBL at the population level, the detailed immunological mechanisms and interactions by which these pathogens facilitate malignant transformation remain unclear.

This narrative review, therefore, aims to synthesize current immunologic and experimental evidence elucidating the mechanisms by which EBV and Pf jointly contribute to the development of eBL. Drawing on findings from reputable research journals, this review seeks to (1) identify and appropriately isolate key interactive innate and adaptive immune pathways through which EBV and Pf influence eBL pathogenesis, and (2) propose a theoretical integrated mechanistic model between EBV and Pf involving critical effector molecules and cytokine groups, leveraging recent, unexplored, or disputed immunological cascades from former studies. This comprehensive analysis aims to improve understanding of the interactions among these pathogens while incorporating speculative discourse that may guide future mouse-model and population-based studies, and further inform novel strategies for early intervention, prevention, and therapeutic targeting in high-burden regions.

### Article selection

**Fig. 1:**
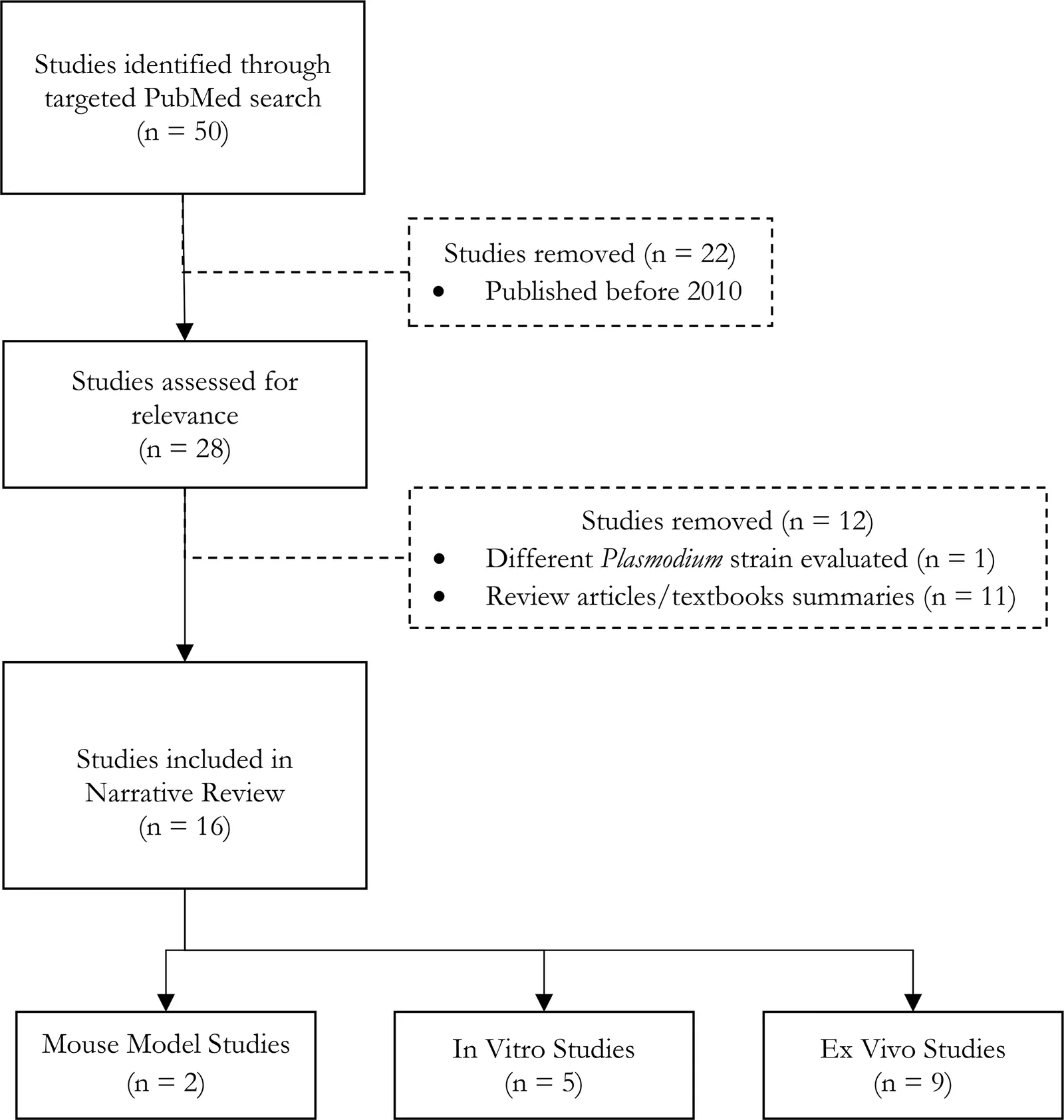
Overview of article identification and selection. Flowchart illustrating the literature selection process. Studies were identified through targeted PubMed searches using predefined key terms and chosen based on their relevance to the review objectives and methodological quality. Studies published before 2010, review articles, textbook summaries, and research on other *Plasmodium* strains were excluded. The final selection included 16 research studies: 2 mouse model studies, 5 in vitro studies, and 9 ex vivo studies. Mouse model studies were defined as experiments evaluating tumor cell lines or treatment protocols directly in mice. In vitro studies were performed in controlled laboratory settings with established eBL or other relevant cell lines. Ex vivo studies involved collecting peripheral blood samples from patients in sub-Saharan Africa (SSA) and analyzing those samples

This review was conducted as a narrative review rather than a systematic review. Given the narrative structure, all reviewed research articles were sourced directly from the academic database PubMed using targeted search terms relevant to the hypothesis (e.g., “Burkitt Lymphoma” + “*Plasmodium falciparum*” or “Malaria” + “Epstein-Barr Virus” + “Mechanism” or “Serology” or “Mice”). Studies were selected based on their relevance to the immunological mechanisms linking EBV and Pf, their methodology, and their contribution to understanding the pathogenesis of eBL. Following the initial search results, studies published before 2010 were not considered to emphasize modern mechanistic and epidemiological evidence, while further ensuring that the review reflects current understanding. Review articles, textbook-derived summaries, and studies investigating different *Plasmodium* strains were also excluded from this analysis. This process resulted in 16 primary research articles that formed the basis of this narrative review. Among the 16 included studies, 2 utilized experimental mouse models, 5 conducted in vitro analyses of EBV-infected B lymphocytes or eBL tumor samples, and 9 used ex vivo methodologies in high-risk regions (Fig. 1). For simplicity, all studies were classified as ex vivo if the analyses were conducted outside the human body, even though in vitro clinical assays were used to generate the data. Together, the 16 included studies provide complementary experimental and epidemiological evidence that informs current hypotheses regarding the interactions between EBV and Pf in malignancy, with details of the articles, including authorship and principal findings, summarized in Table 1. As a narrative review, no predefined systematic study protocol or formal screening, beyond the guidelines listed here, was implemented, and article selection was guided by each study’s relevance and methodological rigor to this review’s objectives. The studies included in this review are intended to be representative of the current literature, and should not be considered an exhaustive account of all the research conducted in this field.

**Table 1:** Research articles and major findings

Author	Title	Place of Publication	Major Findings
Zauner et al. (2010)	TLR9 triggering in Burkitt’s lymphoma cell lines suppresses the EBV BZLF1 transcription via histone modification	*Oncogene*	TLR9, a sensor in B cells which suppresses BZLF1 and prevents EBV from reaching its lytic stage, can be mimicked by *P. falciparum* through releasing hemozoin. This can keep EBV latent.
Kovalchuk et al. (2012)	Mouse model of endemic Burkitt translocations reveals the long-range boundaries of Ig-mediated oncogene deregulation	*Proceedings of the National Academy of Sciences*	In the absence of AID and UNG, sBL tumors were delayed or prevented entirely, likely requiring AID as a primary mechanism for development. However, MYC translocations resembling eBL were found in AID deficient mice, implying a potential alternative mechanism.
Chattopadhyay et al. (2013)	Holoendemic malaria exposure is associated with altered Epstein-Barr virus-specific CD8(+) T-cell differentiation	*Journal of Virology*	While total CMV-specific CD8^+^ T cell populations remain unaffected by holoendemic *P. falciparum* associations, there is a loss of EBV-specific CD8^+^ T cells in children. This can impair immune control function and cause predisposition to eBL.
Torgbor et al. (2014)	A Multifactorial Role for P. falciparum Malaria in Endemic Burkitt’s Lymphoma Pathogenesis	*PLOS Pathogens*	Malaria endemic regions have no major difference in total counts of B cells, however, have increases in GC B Cells which can include EBV-infected cells through enhancing their entry or retention in the GC. *P. falciparum* also can induce deregulated antigen independent AID, hypothesized through hemozoin and PfEMP1, to cooperate with EBV and increase c-MYC translocation risk.
Wilmore et al. (2015)	AID expression in peripheral blood of children living in a malaria endemic region is associated with changes in B cell subsets and Epstein-Barr virus	*International Journal of Cancer*	High frequency malaria transmission regions had elevated AID expression and changes in B cell subsets, which is consistent with increased EBV activity. Malaria may upregulate AID activity and create conditions for increased EBV viral load on the mutated immunoglobulin.
Jordi et al. (2017)	IRAK4 is essential for TLR9-induced suppression of Epstein-Barr virus BZLF1 transcription in Akata Burkitt’s lymphoma cells	*PLOS One*	EBV hijacks the TLR9-MyD88-IRAK4 pathway to maintain latency, a characteristic shared with other gamma herpesviruses, through post-translational modifications and is mostly protein synthesis independent.
Grande et al. (2019)	Genome-wide discovery of somatic coding and noncoding mutations in pediatric endemic and sporadic Burkitt lymphoma	*Blood (American Society of Hematology)*	EBV-positive BL accrue fewer driver mutations specifically in apoptotic genes in order to suppress apoptosis and promote mutagenesis in AICDA and MMR signatures, primarily relying on EBV gene expression for proliferation.
Coghill et al. (2020)	The association between the comprehensive Epstein-Barr virus serological profile and endemic Burkitt lymphoma	*Cancer Epidemiology*,* Biomarkers & Prevention*	Children with eBL showed elevations in IgG, independent of malaria, targeting EBV proteins related to viral replication and anti-apoptosis, while IgA response was unaffected. Authors hypothesize that ongoing EBV replication, implying an impaired T cell response, can increase the number of impacted B cells and increase the risk of eBL.
Forconi et al. (2020)	A New Hope for CD56^neg^CD16^pos^ NK Cells as Unconventional Cytotoxic Mediators: An Adaptation to Chronic Diseases	*Frontiers in Cellular and Infection Microbiology*	Repeated *P. falciparum* infections alongside EBV exposure increase the prevalence of CD56^neg^CD16^pos^ NK Cells. These cells prioritize antibody dependent cytotoxicity for killing antibody opsonized targets, which targets *P. falciparum* (iRBC). However, due to the strong downregulation of multiple cytotoxic and activation receptors, these NK cells poorly target eBL tumors for direct killing, needing to rely on other killing pathways.
Kirimunda et al. (2020)	Variation in the Human Leukocyte Antigen system and risk for endemic Burkitt lymphoma in northern Uganda	*British Journal of Haematology*	No association between eBL and malaria associated HLA types were found, but novel associations in decreased eBL risk with HLA-A and HLA-B type alleles.
Muriuki et al. (2021)	Association of killer cell immunoglobulin-like receptors with endemic Burkitt lymphoma in Kenyan children	*Scientific Reports (Nature)*	Children with eBL have a higher likelihood of carrying over 4 activating KIR genes, hypothesizing that certain patterns of NK cell receptors can increase eBL risk. Additionally, higher EBV viral loads in specific KIR haplogroups imply KIR-mediated NK cell stimulation may impair EBV control, which can add to eBL pathogenesis within malaria and EBV coinfection.
Manara et al. (2022)	Epigenetic Alteration of the Cancer-Related Gene TGFBI in B Cells Infected with Epstein-Barr Virus and Exposed to Aflatoxin B1: Potential Role in Burkitt Lymphoma Development	*Cancers*	Exposure to aflatoxin and EBV infection downregulates TGFBI in B cells via methylation of DNA, allowing EBV to promote immune evasion and unmitigated proliferation.
Saikumar Lakshmi et al. (2023)	Endemic Burkitt lymphoma avatar mouse models for exploring inter-patient tumor variation and testing targeted therapies	*Life Science Alliance*	eBL cell lines and avatar mice models accurately reflect real variations of MYC translocations and EBV status amongst eBL. Additionally, prior to malignant transformation the EBV infected cells may transition through multiple latent profiles.
Burnet et al. (2023)	Hemin treatment drives viral reactivation and plasma cell differentiation of EBV latently infected B cells	*PLOS Pathogens*	The hemin released by *P. falciparum* associated hemolysis can drive the reactivation of EBV as well as inducing degradation of BACH2 through plasma cell differentiation in latently infected memory B cells, and weakening repression of BLIMP1 dependent plasma cell programs.
Muriuki et al. (2023)	Evaluation of KIR3DL1/KIR3DS1 allelic polymorphisms in Kenyan children with endemic Burkitt lymphoma	*PLOS One*	No significant associations were found between the KIRDL1 or KIR3DS1 alleles and eBL risk or EBV viral load, hypothesizing that these NK receptors do not independently impact eBL pathogenesis.
Ariera et al. (2025)	CD4 and CD8 T-cell response is dominated by IL-10-secreting cells in children with uncomplicated Plasmodium falciparum malaria	*ImmunoHorizons*	Acute *P. falciparum* infections in children induces an immunosuppressive T cell dominated by IL-10 secretion and suppression of IFN-𝛾-producing cells. This global T cell dysfunction impairs responses to multiple viral antigens and can provide a mechanism for repeated malaria to weaken immune control of EBV.

## Main text

### Mouse-model studies

The experimental mouse model studies included in this review primarily assessed the oncogenic properties of eBL, with particular emphasis on chromosomal translocation patterns, the presence of EBV and its latent gene expression programs, and the associated immune signaling pathways. Collectively, these experimental protocols provide mechanistic insight into upstream and downstream molecular events that are difficult to disentangle in human observational studies. In the study conducted by Saikumar Lakshmi et al. (2023), eBL tumor biology was evaluated using a combination of patient-derived samples, “long-established, standard” eBL cell lines, and in vivo mouse avatars. Tumor samples obtained from five patients in Kisumu, Kenya, were cultured ex vivo, and a small subset was directly engrafted into immunodeficient (NSG) mice to produce avatar tumors that retained patient-specific disease characteristics (e.g., mass site of origin). The samples produced were compared with standard eBL cell lines (e.g., Raji, Mutu, Akata) and patient-derived cell lines to determine EBV gene expression patterns and therapeutic responses to Rituximab, a key component of eBL treatment [38]. Across all three groups, EBV expression was distinctly heterogeneous, with all tumor cell lines displaying features of multiple EBV latency programs, rather than being restricted to the latency I program. Within this experimental research, these findings challenge the prevailing view that eBL uniformly maintains a restricted latency I transcriptional profile, as reported in earlier studies [23, 33, 34], and instead suggest that EBV-infected B cells may transition through multiple latent profiles prior to malignant transformation. Although Pf was not directly investigated in this study, the observed heterogeneity in EBV latency profiles suggests that distinct latent states may respond differently to Pf-induced immune activation; however, this hypothesis remains speculative and requires direct experimental investigation.

Following Rituximab treatment in the study, the authors further identified biological differences among tumor samples. Resistance or incomplete response to therapy was associated with enhanced type I interferon (IFN) signaling and downregulation of CD20 expression in eBL tumor cells, the receptor necessary for Rituximab binding [38]. Type I IFNs are central mediators of innate antiviral immunity, enhancing antiproliferative activity and recruiting effector immune cells, including natural killer (NK) cells, macrophages, and cytotoxic T lymphocytes. In B lymphocytes, type I IFN signaling also downregulates Bcl-6 expression, a transcription factor required for GC maintenance and B cell survival during affinity maturation [39]. Despite its antiproliferative capabilities, recurrent IFN signaling can induce cellular stress that ultimately creates a microenvironment that paradoxically promotes tumor persistence, immune evasion, and, as observed in this study, therapeutic resistance. Similar IFN-associated immune responses, although limited, have been documented elsewhere,^39^ where chronic signaling was associated with immune states that may support a tumor-supportive environment; however, these studies do not directly establish whether chronic IFN signaling is driving eBL development or is contributing to EBV persistence in the setting of a Pf co-infection. Thus, although this study provides a robust understanding of EBV heterogeneity and the complexities of therapeutic responses, it remains an important question for future investigation whether immune activation driven by Pf infection modifies IFN-signaling cascades, or influences EBV latency states relevant to eBL.

Complementing these findings, Kovalchuk et al. (2012) examined patterns of chromosomal translocations in both sBL and eBL tumor samples from mice predisposed to lymphoma [40]. Given the established role of activation-induced cytidine deaminase (AID) and uracil-DNA glycosylase (UNG) in antibody maturation and diversity during B cell activation in GCs, otherwise known as somatic hypermutation (SHM) and class-switch recombination (CSR), the authors sought to evaluate whether MYC translocations could arise in their absence, and further determine whether those translocations were equivalent to those expressed in human eBL and sBL tumor samples. In light of this, Bcl-xL transgenic mice deficient in AID and UNG were treated with pristane to induce chronic inflammation and subsequent tumor formation. While the absence of AID and UNG resulted in significant delays and, in some instances, the complete prevention of sBL-like tumors, this association was not observed for eBL-like tumors; rather, eBL tumors still occurred, with samples exhibiting variable, nonrandom chromosomal translocations. These discoveries suggest that MYC translocations in eBL can occur through alternative, AID-independent mechanisms. Although previous studies have demonstrated a strong association between aberrant AID activity and MYC translocations,^41,42^ this work indicates that alternative molecular pathways may also contribute to the genomic instability observed in eBL. The specific mechanisms responsible for these chromosomal translocations remain unresolved and show no direct association with Pf.

Together, these mouse model studies highlight the intricacies of BL pathogenesis and identify multiple mechanisms of action that likely contribute to malignancy, though further investigation is needed to understand their roles. The observed heterogeneity in EBV latency, enhanced immune signaling, and the formation of random MYC translocations indicative of eBL are highly suggestive of additional, covert, and advanced mechanisms at play. Because neither study evaluated the effects of Pf infection, any potential role of malaria in modulating these pathways remains strictly hypothetical. The discoveries from these studies may instead provide mechanistic frameworks that inform the interpretation of subsequent in vitro and ex vivo studies. The mouse model data provide biological context for generating hypotheses regarding EBV-Pf interactions but do not establish causal mechanisms underlying human eBL pathogenesis.

### In vitro studies

Studies using in vitro analyses have investigated the interactions between Pf and EBV, focusing on the immunological mechanisms and epigenetic shifts that likely promote the longevity of EBV-infected B cells and contribute to a microenvironment necessary for cancer initiation. Although these experimental studies provide valuable mechanistic insight, their findings should be interpreted in the context of cultured cell models under controlled conditions and not assumed to directly reflect human disease.

**Fig. 2:**
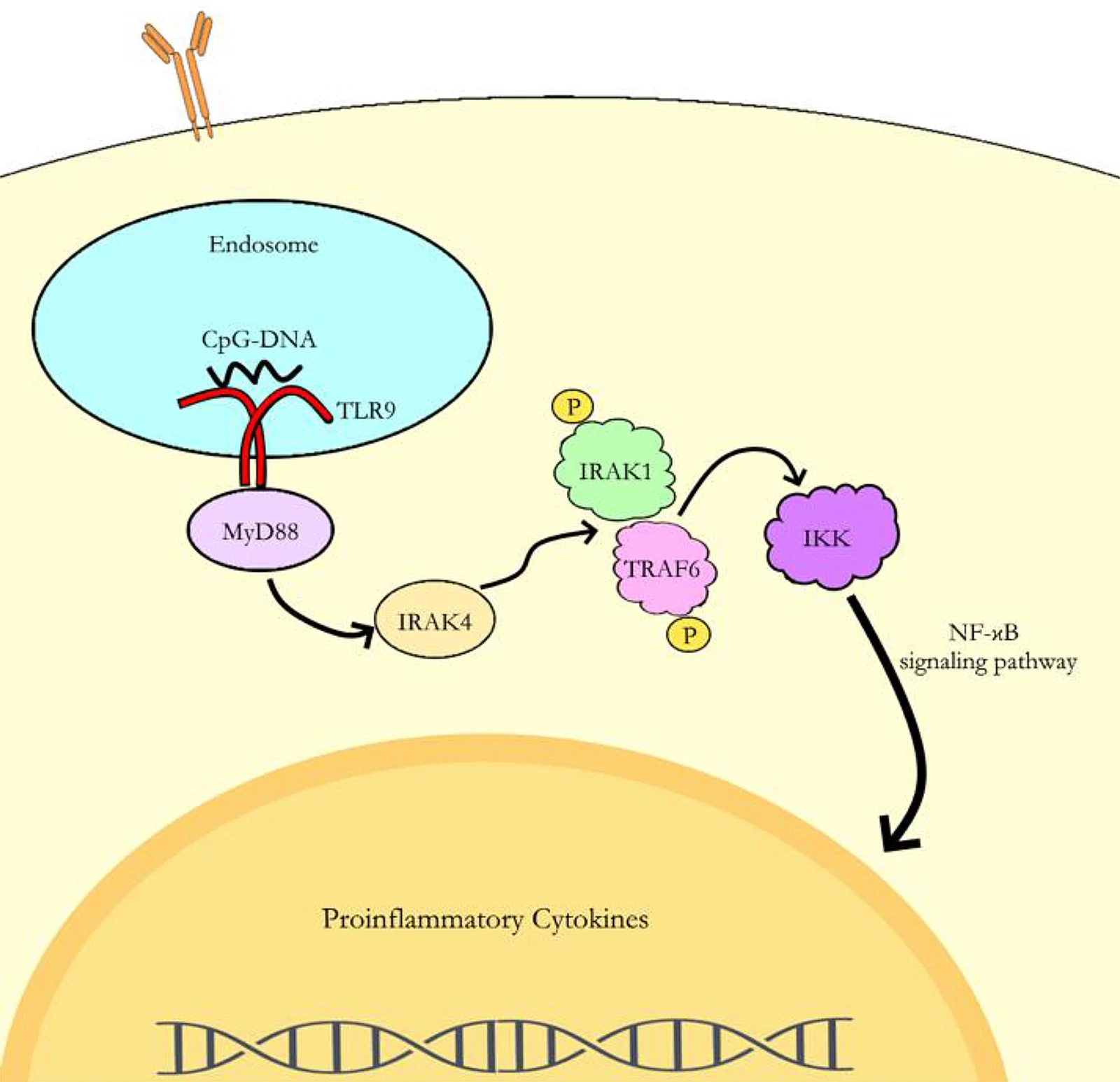
Schematic of the TLR9-MyD88 signaling pathway in B lymphocytes

Zauner et al. (2010) examined the role of Toll-like receptor 9 (TLR9) signaling in EBV latency and the potential exploitation of this pathway by Pf through its crystalline waste product, hemozoin [43]. TLR9, an innate immune receptor located in endosomal compartments, detects unmethylated cytosine-guanine (CpG) motifs commonly found in bacterial and viral DNA. Within B lymphocytes, TLR9 works in concert with B cell receptors (BCRs), which internalize nucleic acids for subsequent presentation to TLR9. Upon CpG detection, TLR9 initiates a MyD88-dependent kinase cascade, leading to the activation of NFκB and the expression of proinflammatory cytokines in the nucleus (Fig. 2) [44]. Using cultured eBL cell lines harboring EBV (e.g., Mutu), the study evaluated the effect of activating and inhibiting TLR9 signaling on EBV expression. Results demonstrated that activation of the TLR9 pathway, using synthetic CpG motifs, suppressed production of BZLF1, EBV’s master regulatory lytic gene. Conversely, inhibiting the TLR9 pathway led to robust gene expression of BZLF1 [43]. Pf’s byproduct, hemozoin, which similarly triggered these shifts in EBV gene expression, did so independently of BCRs and the downstream transcription factor NFκB. These findings suggest that Pf-derived hemozoin may influence EBV latency by modulating TLR9-associated signaling pathways. Whether this mechanism operates during natural Pf infection or contributes directly to human eBL pathogenesis remains unknown. If similar signaling occurs in vivo, then prolonged maintenance of EBV latency could increase the period during which infected B lymphocytes accumulate oncogenic mutations.

Building on the outcomes from Zauner et al., Torgbor et al. (2014) sought to investigate the role of AID in EBV-Pf interactions. To evaluate this, tonsillar B lymphocytes were incubated with Pf under various conditions, and AID expression was measured both directly and through manipulating the TLR9 pathway [45]. Findings showcased that while the presence of Pf alone induced minimal AID expression, the combination of Pf, Interleukin-4 (IL-4), and CD40 ligand (CD40L) significantly increased AID expression, indicating that helper (CD4+) T lymphocytes were a necessary component in stimulating AID expression in the presence of Pf [41, 42]. The presence of hemozoin in the intubated B lymphocytes additionally promoted AID expression, particularly when BCR and TLR9 pathways were simultaneously activated. This interaction was interpreted as evidence of functional crosstalk between BCR and TLR9 [44], potentially mediated by an additional Pf component, namely PfEMP1. Finally, a comparison of GC tonsillar B cells infected with Pf and controls showed that Pf-infected cells had a markedly higher EBV load, approximately 70-fold higher than that of their counterparts. Overall, the study highlighted a potential synergistic interaction in which Pf enhances AID expression in response to appropriate immune stimulation, while EBV promotes the persistence of genetically altered B cells. Since these findings were observed with cultured B lymphocytes, further studies are required to determine whether similar interactions occur during natural Pf infection.

Jordi et al. (2017) further dissected the TLR9-MyD88 pathway and its role in EBV latency and lytic reactivation using eBL cell lines known to remain latent (e.g., Akata). [46] The authors directly stimulated BCRs and corresponding TLR9 receptors, and utilized CRISPR/Cas9 to knock out key downstream kinases as a means to determine which aspects of the pathway were exactly impacting EBV expression patterns. The kinases investigated included IRAK4 and IRAK1, as depicted in the TLR9-MyD88 schematic (Fig. 2). The loss of TLR9, MyD88, or IRAK4 was shown to disrupt TLR9-mediated suppression of EBV lytic reactivation, resulting in the increased transition from latent to lytic EBV following BCR activation. Interestingly, IRAK1 knockout resulted in the complete loss of the EBV genome, suggesting that IRAK1 plays a critical role in viral genome maintenance regardless of latent or lytic status. These results support Zauner et al.’s conclusions and further support the hypothesis that the TLR9-MyD88-IRAK4 signaling axis contributes to the maintenance of EBV latency under controlled settings. Further research evaluating whether modulation of this pathway could produce similar effects in vivo or provide an effective therapeutic strategy is thus warranted.

Continuing the exploration of Pf byproducts, Burnet et al. (2023) investigated the role of hemin, a toxic metabolite produced by Pf following hemoglobin digestion, on EBV gene expression. In the study, EBV-infected eBL cell lines (e.g., Mutu I) were treated with varying doses of hemin, and gene expression patterns were evaluated by RT-qPCR at different time points [47]. Unlike the latency-promoting effects of hemozoin reported in other in vitro studies,^43,45^ hemin exposure in this study was associated with increased expression of BZLF1, the transcription factor that initiates EBV lytic reactivation. The direct pathway with which this occurred, however, was not completely understood. RNA sequencing further revealed the upregulation of genes essential for plasma cell differentiation, a specialized form of B cells that exclusively produce and secrete antibodies necessary for combating infection. These transcriptional changes were accompanied by reduced expression of BTB and CNC homolog 2 (BACH2), a transcription factor that primarily suppresses premature plasma cell differentiation in activated B cells [48]. The authors proposed that the loss of BACH2 may facilitate EBV reactivation and promote infection of neighboring B cells, though further investigation is needed to establish this causal pathway in vivo. Collectively, these findings suggest that Pf-derived hemin may promote EBV lytic reactivation through BACH2-associated pathways; this discovery provides a potential mechanism between Pf and EBV that requires further analysis in natural, uncontrolled settings.

In contrast, Manara et al. (2022) evaluated the combined effects of EBV and the environmental carcinogen Aflatoxin B1 (AFB1) on epigenetic shifts in cultured B cells [49]. Their work directly involved exposing B cells to AFB1 and EBV and monitoring DNA methylation events through methylome analysis and RNA sequencing. DNA methylation is an epigenetic modification in which a methyl group is added to DNA, leading to transcriptional repression [50] .Results from the study demonstrated that long-term exposure to both AFB1 and EBV increased DNA methylation at the promoter of the transforming growth factor beta-induced (TGFBI) gene, a critical regulator of cell proliferation and apoptosis. The complete deletion or silencing of TGFBI, as illustrated in this study, has been shown to induce tumor progression and subsequent distant metastasis [49, 51]. While AFB1 alone was insufficient to fully repress TGFBI, EBV exposure completed this epigenetic silencing, suggesting that persistent EBV infection may contribute to a permissive epigenetic environment that can be further modified by other cofactors. While this study does not examine the role of Pf and EBV in B cell epigenetics, these results provide broader context for understanding how multiple environmental exposures may cooperate with EBV to promote oncogenic transformation.

These in vitro studies have suggested several mechanisms through which EBV and Pf may interact to influence B-cell biology. Across the different experimental systems, Pf byproducts, namely hemozoin and hemin, in tandem with Pf antigens, have been reported to alter signaling pathways, specifically TLR9 and BCR signaling, increase AID expression, reinforce EBV latency under some experimental conditions, and promote expression of EBV lytic genes under others. These molecular changes have been proposed to further enhance the persistence, dissemination, and genomic instability of EBV-infected B cells, although the extent to which these mechanisms directly contribute to eBL development in humans remains to be established. Furthermore, persistent EBV infection has been associated with epigenetic alterations, including those influenced by environmental carcinogens, that suppress key tumor suppressor genes, potentially fostering a microenvironment conducive to oncogenic transformation. Altogether, these experimental findings offer valuable mechanistic insights into how Pf and EBV may interact at the cellular level; however, additional validation in animal models and human studies is necessary to determine their direct relevance to eBL pathogenesis.

### Ex vivo studies

Of the ex vivo studies included in this review, one study by Grande et al. (2019) focused primarily on the genomic characteristics of EBV-positive eBL, rather than on specific immune effector or cytokine responses, while the eight remaining studies primarily examined immunological features associated with EBV and Pf infection and their potential contributions to eBL pathogenesis. These eight studies collectively evaluated five immunological domains: Human Leukocyte Antigen (HLA) molecule expression, AID expression, T-cell responses, antibody-mediated humoral responses, and NK cell receptor expression.

As noted, Grande et al. conducted a comprehensive genomic investigation to explore the mutational landscape of EBV-positive BL and to determine whether genomic differences existed between endemic and sporadic forms of the disease. Archived EBV-positive eBL and sBL tumor samples from Uganda and the U.S., obtained directly through the Burkitt Lymphoma Genome Sequencing Project (BLGSP) and the International Cancer Genome Consortium, underwent whole-genome sequencing [52]. Results revealed that, although all BL samples require the MYC translocation, EBV-positive tumors, irrespective of geographic location, had fewer classic driver mutations, with EBV-positive sBL tumors closely resembling eBL tumors at the genomic level. The most common mutational signatures observed in the EBV-positive tumor samples were consistent with defects in the mismatch repair (MMR) pathway, a DNA repair mechanism responsible for correcting base-pairing errors introduced during DNA replication [53], potentially induced through epigenetic silencing [49, 50]. Additionally, noncoding mutations were observed almost exclusively in EBV-positive tumors and were enriched at AID-targeting motifs. Based on these findings, the authors proposed that EBV may confer functional advantages that reduce the requirement for additional driver mutations during tumor development, potentially through mechanisms that promote immune evasion or inhibit apoptosis. While the mechanisms were not directly illustrated in this study, the genomic data support the possibility that EBV contributes to lymphomagenesis through pathways that extend beyond MYC translocations alone. Because Pf was not explicitly examined in the genomic analysis, the study’s findings still complement previous experimental studies suggesting that EBV possesses intrinsic oncogenic potential. When considered alongside evidence from in vitro models examining Pf-associated immune dysregulation, these results support the hypothesis that Pf may act as a cofactor that enhances, rather than initiates, EBV-driven eBL development. Additional studies that directly evaluate both pathogens in human tissues are needed to clarify this proposed interaction.

While Grande et al. highlight EBV’s genomic contributions to eBL progression, a complete understanding of tumor development requires a comprehensive examination of effector-pathogen interactions. The following subsections summarize the eight effector- and humoral-immune-focused ex vivo studies, organized by the five previously identified key domains, to better understand how EBV-Pf interactions are likely to shape the immune landscape.

#### HLA molecule expression

The adaptive immune response is largely regulated by HLA molecules, which present self- and non-self antigens on the cell surface as part of the major histocompatibility complex (MHC), enabling T lymphocytes to recognize and respond to foreign pathogens [54]. HLA molecules can be grouped into two main classes: Class I (HLA-A, HLA-B, HLA-C), which are expressed on the surface of all nucleated cells and present intracellular antigens to cytotoxic (CD8+) T cells, and Class II (HLA-DP, HLA-DQ, HLA-DR), which are expressed on antigen-presenting cells (APCs) such as macrophages, B cells, and dendritic cells and present extracellular antigens to CD4 + T cells [54] .Through this intricate system, HLA molecules play a critical role in fighting infection by coordinating effective immune responses, serving as the bridge that enables robust communication between effector and infected cells. Given their central role in antigen presentation, HLA variation represents an important area of investigation for understanding host susceptibility to EBV, Pf, and eBL.

Kirimunda et al. (2020) thus investigated whether HLA allelic variation was associated with eBL risk among EBV-positive children from Northern Uganda with a history of current, past, or recurrent Pf infection [55]. Using high-resolution next-generation sequencing of HLA genes from 200 eBL cases and 400 population-based controls (aged 1–15 years), the investigators identified three main HLA Class I alleles (HLA-A*02, HLA-B*41, and HLA-B*58) that were strongly associated with reduced eBL risk. Conversely, homozygosity for the HLA Class II allele HLA-DQA1 was strongly associated with increased eBL risk. The authors suggested that reduced diversity in antigen presentation may impair CD4 + T cell activation by APCs, thereby weakening adaptive immune responses. If antigen presentation is compromised, persistent infections such as EBV and Pf may be less effectively controlled, potentially facilitating immune evasion and prolonged pathogen persistence. Although the study did not directly assess immune function or pathogen control, the observed genetic associations are consistent with the hypothesis that HLA variation may influence host responses to EBV and Pf. Overall, the findings from this study suggest that differences in HLA genotype may contribute to variation in eBL susceptibility, although additional functional studies are needed to determine the underlying immunological mechanisms.

#### AID expression

As discussed in previous sections, AID has been repeatedly implicated in eBL pathogenesis, with experimental studies suggesting that recurrent Pf exposure may promote aberrant AID activity, thereby influencing EBV persistence [41, 42, 45]. Building on these findings, Wilmore et al. (2015) examined whether chronic Pf exposure was associated with altered B-cell populations, EBV viral load, and AID expression in two cohorts of 3-year-old children from two locations in rural Kenya: the Kisumu District (*n* = 54), a high malaria transmission region, and the Nandi District (*n* = 34), a low malaria transmission region [56]. Peripheral mononuclear cells were analyzed via flow cytometry to characterize B cell subsets, while qPCR was used to measure EBV viral load and AID mRNA expression. The study discovered significant changes in memory B cell populations associated with high-frequency Pf exposure. Specifically, children from the Kisumu District had fewer immature/transitional B cells and immunoglobulin M (IgM) memory B cells, and instead displayed increased atypical (exhausted-state) B cells, consistent with prior observations in malaria-endemic populations [35–37]. Additionally, all samples showed detectable AID expression in the blood, with the authors noting that the elevation was likely consistent with chronic B cell activation. While the study did not directly establish that recurrent Pf infection induced these immunological changes, the findings support an association between sustained malaria exposure and altered B-cell activation in young children. These ex vivo observations are broadly consistent with previous in vitro studies, including those by Torgbor et al., which demonstrated that Pf-derived components can stimulate B-cell activation through TLR9 signaling pathways. However, because the two studies employed different experimental systems, the mechanistic relationship between chronic Pf exposure, AID dysregulation, and EBV persistence cannot be directly inferred from these data alone. Taken together, the available evidence supports the hypothesis that repeated exposure to Pf may contribute to prolonged B-cell activation, creating conditions that favor EBV persistence and increase the risk of genomic instability associated with eBL.

#### T cell responses

To better understand the key effector cells that may contribute to eBL pathogenesis, it is essential to analyze T lymphocyte subsets. Within the adaptive immune response, CD4 + T cells support B cell activation and further help to activate, modulate, and maintain CD8 + T cell responses, while CD8 + T cells directly eliminate infected cells by recognizing antigens presented on Class I molecules [54, 57]. Given their role in combatting infections, alterations in T-cell function may influence host responses to both EBV and Pf. Using a similar population-based approach to Wilmore et al., Chattopadhyay et al. (2013) aimed to evaluate whether chronic Pf exposure was associated with changes in EBV-specific CD8 + T cell populations. Peripheral blood samples from children without eBL living in the Kisumu and Nandi Districts were analyzed to compare T cell phenotypes between regions of high and low malaria transmission [58]. The study found that children living in areas of chronic Pf exposure still produce EBV-specific CD8 + T cells; however, these cells exhibited altered phenotypic characteristics. In particular, central memory EBV-specific CD8 + T cells were less frequent in children from the high-transmission region. The authors suggested that this reduction reflected progressive differentiation rather than deletion of the T cell population, which could potentially diminish long-term immune control of EBV. Most importantly, the association between chronic Pf exposure and reduced EBV-specific memory T cells remained even after adjusting for age. The study’s conclusions suggest that recurrent Pf infections may influence the maintenance of EBV-specific T cell immunity, although, given the study’s experimental approach, the mechanisms underlying these shifts were not investigated.

To further characterize T cell responses during malaria, Ariera et al. (2025) examined immune cell profiles in pediatric patients with uncomplicated Pf malaria treated at the Chulaimbo Sub-County Hospital in western Kenya [59]. Peripheral blood samples were collected from 10 infected children and 10 geographically matched controls and subsequently analyzed by flow cytometry. Results showed that, although overall T cell activation profiles were largely unchanged in children with Pf infection compared with controls, cytokine production differed significantly between the groups. Specifically, both CD4 + and CD8 + T cells in Pf-infected children showed higher frequencies of IL-10-secreting cells. IL-10, produced by various immune cells, serves as an immunoregulatory cytokine that “impairs proliferation, cytokine production and migratory capacities of effector T cells” [60]. In contrast, the frequency of IFN-γ-producing T cells, which normally drive antiviral and pro-inflammatory immune responses [61], was significantly reduced. Notably, these drastic cytokine changes were not EBV-specific, as similar responses were observed following stimulation with cytomegalovirus antigens. The authors therefore concluded that uncomplicated Pf malaria is associated with a broader shift toward a more immunoregulatory T cell profile rather than an EBV-specific impairment.

Both the Chattopadhyay and Ariera studies provide critical insights into how repeated exposure to Pf shapes T cell populations in the context of EBV. Chattopadhyay et al. identified changes in EBV-specific memory CD8 + T cell populations in children from malaria-endemic regions, and Ariera et al. described broader immunoregulatory changes characterized by increased IL-10 production and reduced IFN-γ secretion. Although these studies did not directly examine the effects of these immune changes on EBV persistence or eBL development, their findings are consistent with experimental evidence suggesting that chronic malaria exposure may create an immune environment that is less effective at controlling latent viral infections. Further studies integrating immunological and clinical data are needed to determine exactly how these T cell modifications contribute to EBV persistence and the subsequent risk of eBL.

#### Antibody-mediated humoral responses

Humoral immunity is an important component of host defense against both EBV and Pf and may provide valuable insight into the immunological changes associated with eBL. Produced directly by differentiated plasma cells, antibodies mediate protection against infection through multiple mechanisms: direct neutralization of infected cells, opsonization facilitating phagocytosis by macrophages, antibody-dependent cellular cytotoxicity (ADCC) mediated by NK cells, and complement-mediated pathogen lysis [62]. Given their functional versatility and their reflection of both prior antigen exposure and ongoing immune activity, characterizing EBV-specific antibody profiles may help identify patterns associated with altered viral control in individuals with eBL. Coghill et al. (2020) explored EBV-specific antibody responses in children with eBL and healthy controls in Ghana, using serum samples collected by the U.S. Cancer Institute between 1965 and 1994 [63]. Their analysis involved directly measuring EBV-specific immunoglobulin A (IgA) and IgG levels across the samples. The analysis found that children with eBL exhibited elevated IgG antibody levels compared with controls, whereas IgA responses did not differ significantly. The most pronounced IgG responses were directed against EBV proteins involved in viral replication (BMRF1), lytic activation (BZLF1), virion assembly (BBLF1), and apoptotic inhibition (BHRF1), while antibody responses to latent EBV proteins were comparatively lower. These results likely suggest that eBL is associated with a distinct serologic profile characterized by enhanced antibody responses to lytic EBV antigens. After adjusting for Pf exposure, the association between elevated EBV-specific IgG responses and eBL remained, signifying that these antibody profiles were not solely explained by malaria exposure. The authors thus proposed that Pf may contribute to eBL through immune modulation and B cell activation rather than acting as an independent driver of oncogenesis. Overall, Coghill’s results suggest that altered humoral responses to EBV may serve as immunological markers associated with eBL. Because the study was observational, it is difficult to establish whether these antibody patterns contribute to malignant transformation or rather reflect changes that occur during disease development. Nonetheless, when considered alongside experimental studies demonstrating Pf-associated immune dysregulation, these findings are consistent with the hypothesis that chronic Pf exposure may modify host immune responses to EBV in ways that influence eBL susceptibility.

#### NK cell receptor expression

The mechanisms by which NK cells might influence or exacerbate oncogenic processes in eBL settings remain relatively underexplored in the literature. Only recently have researchers begun to examine how NK cells respond to chronic pathogen exposure in the context of those that drive eBL-permissive environments. In their natural state, NK cells function within the innate immune system, directly killing infected or tumor cells by releasing cytotoxic lytic granules or indirectly via antibody-dependent mechanisms. In addition to these functions, NK cells secrete cytokines that enhance innate immune responses and further shape adaptive immunity [64]. To adequately perform these functions, NK cells rely on a variety of surface proteins that facilitate target cell recognition, cellular adhesion and synapse formation, and direct engagement with antibodies. The two key surface receptors involved in these processes are CD56, which mediates direct killing, and CD16, which mediates antibody-mediated killing (ADCC). While not true selective markers of NK cells, these surface receptors allow NK cells to be categorized as high-expressing (positive), low-expressing (dim), or absent/undetectable (negative) [64].

In the study by Forconi et al. (2020), the authors investigated whether CD56^neg^CD16^pos^ NK cells represented a distinct subset of NK cells in individuals with eBL [65]. Historically, NK cells expressing CD56^neg^ were either misclassified or not recognized as NK cells. Previous literature has also indicated that these subtypes are strongly associated with chronic Pf exposure among eBL patients in SSA. To appropriately characterize these cells, Forconi et al. analyzed peripheral blood samples from 8 eBL cases and 3 healthy controls in Kenya. Transcriptional profiling and morphological analyses demonstrated that CD56^neg^CD16^pos^ cells shared many characteristics with conventional CD56^dim^CD16^pos^ cells, supporting their classification as a legitimate NK cell subset. Although they shared similar transcriptional factor profiles, NK cells expressing CD56^neg^, which were particularly prominent in eBL cases, exhibited upregulation of genes related to antibody-dependent immune functions and downregulation of genes involved in classical NK cell cytotoxicity. The results thus suggest that in regions of holoendemic malaria, chronic Pf exposure may be associated with shifts in NK cell phenotype that favor antibody-dependent functions over direct cytotoxicity activities. The authors note that while this adaptation likely improves NK cell-mediated control of Pf infection, it may simultaneously affect immune surveillance of EBV-infected cells, though this functional relationship was not directly examined in the study.

Complementing these phenotypic observations, Muriuki et al. (2021) examined whether genetic variation in killer immunoglobulin-like receptors (KIRs) was associated with eBL [66]. KIRs are transmembrane receptors expressed on NK cells, distinct from “selective markers,” that interact with HLA Class I molecules to regulate cytotoxic activity through either activating or inhibitory signals [67]. Following KIR genotyping of DNA extracted from peripheral blood mononuclear cells, the authors observed that children with eBL were more likely than controls to carry four or more activating KIR (aKIR) genes, suggesting that this subgroup may have a genetic predisposition toward heightened NK cell activation. Given this association, the researchers hypothesized that increased numbers of aKIR genes may contribute to chronic NK cell activation, potentially sustaining a pro-inflammatory environment or promoting NK cell dysfunction that could impair immune control of persistent viral infections, such as EBV. These proposed mechanisms were not directly evaluated in the study, and additional analyses are required to determine whether these observed genetic associations influence NK cell activity in vivo.

To further investigate the role of inhibitory KIRs (iKIRs), Muriuki et al. (2023) evaluated the inhibitory receptor gene, KIR3DL1, to determine whether iKIR variation was associated with eBL susceptibility [68]. Unlike aKIRs, which promote NK cell cytotoxic activity, iKIRs suppress NK cell activation and help maintain self-tolerance by preventing inappropriate targeting of healthy cells. In this study, DNA samples from 108 eBL patients and 99 healthy controls in Kenya were analyzed to determine the distribution of KIR3DL1 genotypes and EBV viral loads. The results demonstrated that eBL patients exhibited significantly higher EBV viral loads than controls; however, the distribution of KIR3DL1 did not differ significantly between the two groups. These findings suggest that KIR3DL1 alone is unlikely to account for differences in eBL susceptibility, and that aKIRs may play a more prominent role in NK cell dysregulation. The authors noted that additional iKIR genes should be investigated before broader conclusions regarding inhibitory NK cell signaling can be drawn.

Together, these studies suggest that chronic Pf exposure and host genetic variation may be associated with altered NK cell phenotypes and receptor expression in individuals with eBL. The available evidence suggests that these changes may affect immune responses to EBV, though the exact functional consequences for viral control and subsequent lymphoma development remain unclear. Complementary research evaluating NK cell populations using animal models and in vivo data is necessary to elucidate the contribution of NK cells to EBV-Pf interactions and eBL development.

## Discussion

**Fig. 3:**
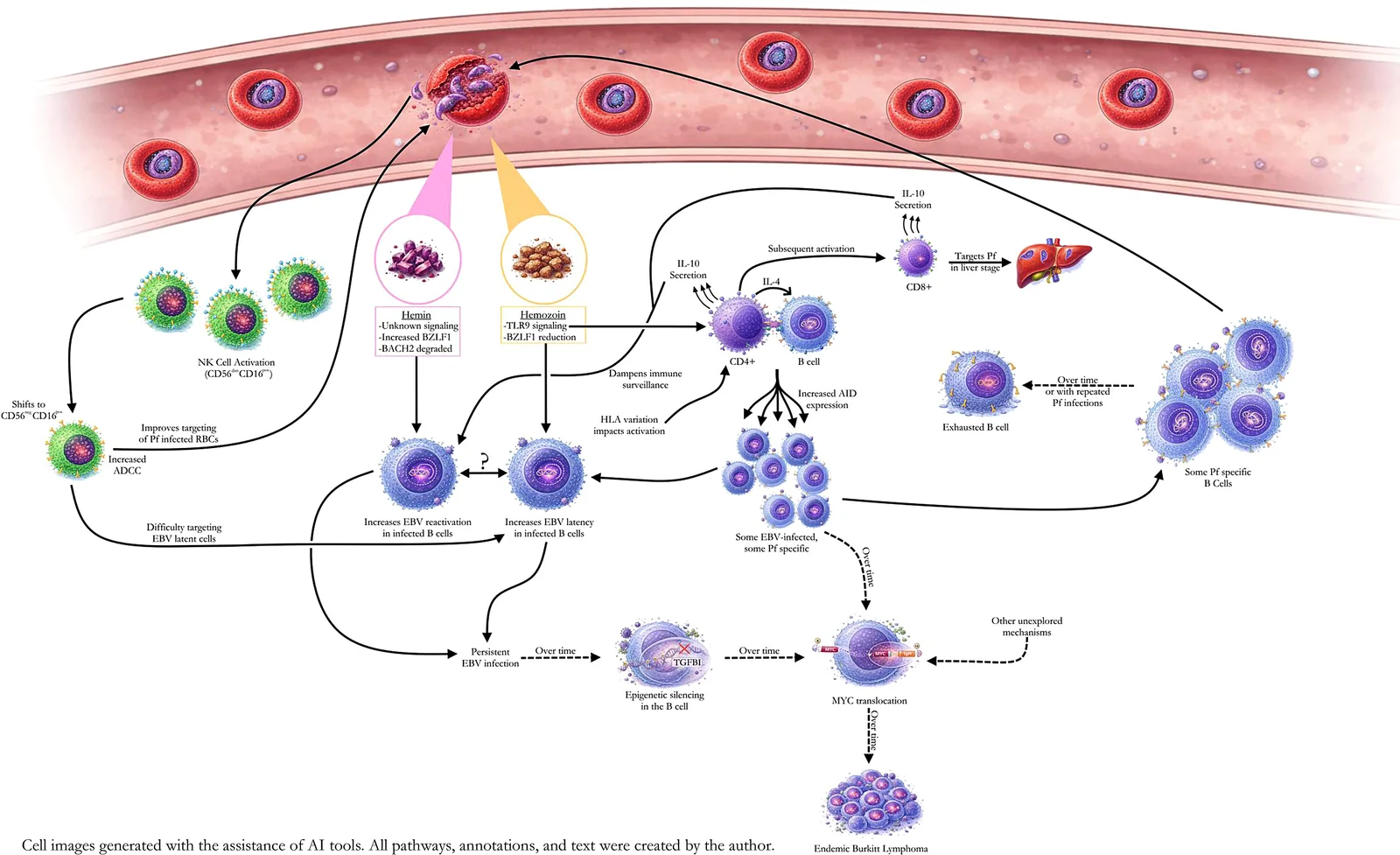
Proposed Multi-Hit Model of EBV-*P. falciparum* Interactions in eBL

This narrative review synthesizes current evidence describing the complex immune interactions between EBV and Pf, with a particular focus on the immune effector cells, signaling pathways, and cytokine responses that may contribute to eBL pathogenesis. Together, the studies reviewed several immunological mechanisms implicated in EBV-Pf interactions. By integrating findings from animal studies, in vitro experiments, ex vivo analyses of human samples, and genomic investigations, this review proposes a theoretical conceptual framework that blends these observations into a model of eBL development (Fig. 3). Because this framework draws on heterogeneous experimental protocols rather than a single, continuous body of evidence, it should be interpreted as a hypothesis-generating model rather than a definitive mechanistic pathway for human disease. The convergence of findings across multiple study types, in this instance, provides a biologically plausible basis for understanding how chronic Pf exposure may influence EBV persistence and contribute to lymphoma development. Rather than operating as isolated risk factors, EBV and Pf are theorized to participate in a recursive immunological loop characterized by repeated immune activation, viral reprogramming, and progressive immune dysfunction.

A salient feature of the proposed model is the ability of Pf-derived byproducts to moderate EBV latency and lytic reactivation through distinct mechanisms. In vitro studies suggested that hemin, a toxic metabolite released during RBC rupture, promotes plasma cell differentiation and is associated with increased expression of the EBV lytic regulator BZLF1 [47]. Burnet et al. further reported the degradation of the transcription factor BACH2 following hemin exposure, proposing that its loss may facilitate EBV lytic reactivation [47, 48]. Although the precise receptor and signaling cascade mediating this hemin-induced change remain unresolved, TLR4 has been proposed as a plausible candidate given its surface expression on B cells and its known interactions with foreign antigens, but this hypothesis has not been directly demonstrated [47]. In contrast, experimental studies examining hemozoin have reported activation of TLR9 signaling accompanied by suppression of BZLF1 expression, findings that are consistent with maintenance of EBV latency under those experimental conditions [43, 46].

Overall, these findings suggest that chronic Pf exposure may simultaneously promote viral persistence and lytic reactivation, depending on the surrounding immunological milieu. The temporal relationship between these opposing stimuli, however, remains unclear. Whether hemin- and hemozoin-mediated signaling occurs sequentially, simultaneously, or in a latency-state-dependent manner remains to be established. This proposed bidirectional process is consistent with findings from Saikumar Lakshmi et al., who demonstrated that EBV-infected B cells exhibited considerable variation across latency programs [38]. Certain latency states, particularly latency III [69], have been shown to be more permissive to lytic reactivation, suggesting that they may respond differently to Pf-associated stimuli, although this hypothesis needs to be investigated under controlled conditions.

Beyond direct modulation of viral gene expression, chronic Pf exposure has also been associated with sustained polyclonal B cell activation and alterations in memory B cell populations [35–37, 56]. Persistent antigenic stimulation is thought to promote the direct recruitment and expansion of CD4 + T cells, which support GC responses through SHM and CSR, processes that depend on AID upregulation. While AID is essential for producing high-affinity antibodies, prolonged or dysregulated AID activity has also been implicated in genomic instability. Experimental and ex vivo studies reviewed here suggest that repeated exposure to Pf may contribute to sustained AID expression, thereby creating conditions that could increase the likelihood of off-target DNA damage in EBV-infected B cells [45, 49, 56]. Within this framework, repeated cycles of proliferation, together with EBV-mediated survival signaling and epigenetic alterations, may increase the probability of oncogenic events such as MYC translocations, the defining molecular hallmark of eBL [1–3]. Importantly, murine studies demonstrating MYC translocations in AID-deficient settings indicate that AID may not be the sole mediator of genomic instability [40]. Additional mechanisms, including oxidative DNA damage, replication stress, or impaired DNA repair, may also contribute to malignant transformation; however, given limited data, these hypotheses require further exploration.

Immune dysregulation extending beyond B cells may further contribute to the permissive environment proposed in this model. Ex vivo studies identified elevated IL-10 production and reduced IFN-γ responses in children with chronic Pf exposure, changes that are consistent with a more immunoregulatory immune profile [58, 59]. Although IL-10 limits excessive inflammation during acute infections, sustained IL-10 signaling may significantly dampen antiviral immunity and potentially influence EBV-specific CD8 + T cell responses. Over time, CD8 + T cells may acquire features of functional exhaustion, analogous to those reported in chronically stimulated B cell populations. Within the proposed conceptual framework, this progressive dysfunction could limit the host’s capacity to control EBV-infected B cells undergoing intermittent lytic reactivation or transitions between latency programs. Similarly, studies of NK cell biology have described changes in NK cell phenotypes associated with chronic Pf exposure [65, 66, 68]. These observations suggest that recurrent Pf infection may alter both innate and adaptive immune responses in ways that directly influence EBV immune surveillance.

Taken together, these interacting mechanisms within the conceptual framework may form a self-reinforcing immunological cycle. Recurrent Pf exposure could promote chronic immune activation and repeated B cell stimulation, while EBV exploits these activated B cells to establish persistence, undergo periodic lytic reactivation, and expand its reservoir of infected cells. Over prolonged periods of co-exposure, cumulative genetic and epigenetic alterations, including MYC translocations, may increase the likelihood of malignancy. This theoretical model provides one biologically plausible explanation for why eBL may develop in regions with intense Pf transmission.

An important limitation of this proposed framework, as previously noted, is its reliance on evidence from multiple experimental systems. Although these complementary approaches have been merged to produce a coherent biological narrative, they do not establish a continuous causal pathway linking EBV and chronic malaria exposure to eBL development in humans. The model should thus be viewed as a synthesis of the current literature in the field, which can be used to generate testable hypotheses, rather than as a definitive description of eBL pathogenesis. Additionally, despite these detailed advances in understanding the mechanisms that intertwine EBV and Pf, several mechanistic gaps remain. For instance, the precise signaling pathway by which hemin promotes EBV reactivation has not been definitively characterized. The temporal dynamics governing EBV transitions between latency and lytic states under Pf-driven immune pressure also seem difficult to model. Finally, the definition and measurement of “exhaustion” across B cells, CD8 + T cells, and NK cells as they undergo transcriptional changes remains variable, complicating the interpretation and direct categorization of functional impairment across effector types. Future studies integrating longitudinal human cohorts with paired mechanistic experiments will be essential for determining whether the interactions proposed herein occur during natural infection and in refining the model of eBL development.

### Expansion of the mechanistic model: emerging effector molecules in EBV-Pf pathogenesis

As the mechanistic framework linking EBV and Pf to eBL progression continues to evolve, incorporating additional immune effector populations may further refine our understanding of eBL. Building upon the evidence synthesized throughout this review, an additional conceptual framework has been proposed in which neutrophils may represent an additional immune cell population capable of influencing EBV-Pf interactions. This proposed mechanism is informed primarily by findings from a recent preprint by Akinyi et al. (2025), as well as the broader literature on neutrophil biology, oxidative stress, and EBV latency. In their study, the phenotypic profiles of neutrophils among Pf-infected children with eBL and healthy controls were evaluated in western Kenya [70]. Results demonstrated that children with recurrent Pf exposure who were subsequently diagnosed with eBL exhibited a significant increase in aged and atypical-aged neutrophil subsets. These findings suggest that chronic exposure to Pf greatly reshapes neutrophil homeostasis toward a sustained pro-inflammatory phenotype. Since these findings are based on a single preprint, additional validation in independent cohorts is required.

Neutrophils are recognized for their antimicrobial functions, including, but not limited to, phagocytosis, degranulation, respiratory bursting, and neutrophil extracellular trap (NET) formation [71]. Recent research on this effector cell type has additionally demonstrated that chronic inflammatory states can reprogram neutrophil populations, promoting subsets with enhanced inflammatory capacity and altered developmental stages [71]. Aged neutrophils, contrary to mature circulating neutrophils, are defined by prolonged circulation, enhanced production of reactive oxygen species (ROS), and generally increased inflammatory potential [72]. ROS are highly reactive oxygen-containing molecules generated during neutrophil respiratory bursts, including superoxide and hydrogen peroxide [71, 72]. Although extremely necessary for antimicrobial defenses, excessive ROS can induce oxidative stress and consequently activate stress response signaling pathways [71, 72].

**Fig. 4:**
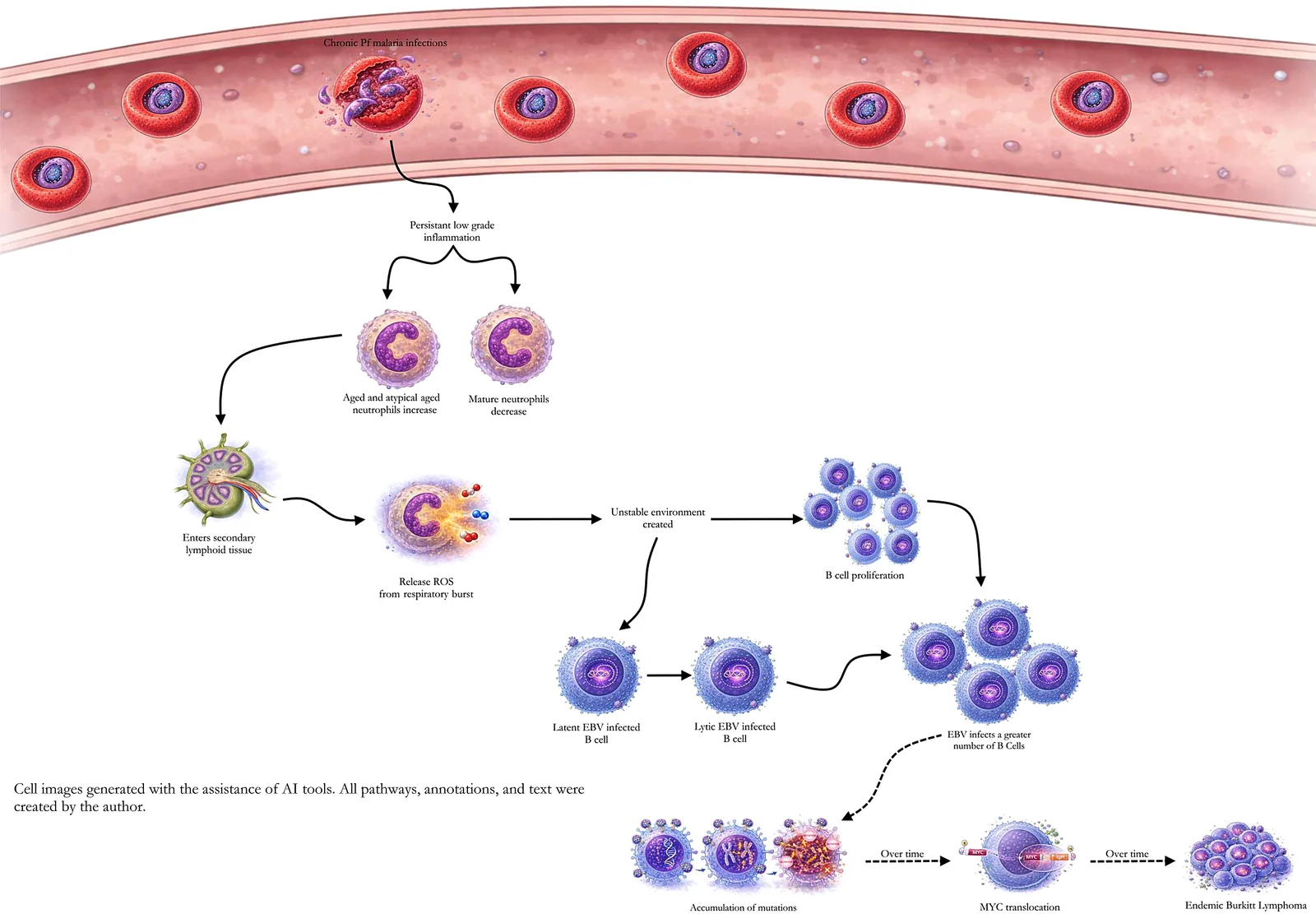
Hypothesized neutrophil-mediated pathway contributing to eBL pathogenesis

Within the proposed conceptual mechanistic framework (Fig. 4), chronic Pf exposure may promote the expansion of inflammatory neutrophil subsets that preferentially accumulate within inflamed secondary lymphoid tissues, anatomical sites where latent EBV persists in memory B cells. Concurrently, the broader immune alterations described throughout this review – including increased IL-10 production, altered EBV-specific CD8 + T cells, sustained B cell activation, and altered NK cell function – may reduce immune surveillance of infected B cells. Thus, in this proposed immunological landscape, neutrophil-derived ROS could generate local oxidative stress and inadvertently activate B cells. In this conceptual framework, repeated cycles of inflammation-driven B cell activation could potentially increase opportunities for genomic instability. This hypothesis does not aim to classify neutrophils as direct oncogenic drivers in eBL pathogenesis. Rather, it proposes that chronic Pf-associated remodeling of neutrophil populations could reinforce the broader immune dysregulation described throughout this review. If experimentally validated, this expanded model would identify neutrophils as additional contributors to the complex immune milieu linking chronic Pf exposure to EBV persistence and eBL development.

## Conclusion

This narrative review synthesizes current evidence into a conceptual framework describing how EBV and Pf may interact to contribute to the pathogenesis of eBL. Collectively, the findings suggest that chronic Pf exposure may do more than simply coexist with EBV; it may reshape the immune landscape in ways that favor persistent viral infection, repeated immune activation, and dysregulated B cell responses. By integrating findings from studies with different experimental systems, this review proposes that eBL may arise as the cumulative consequence of persistent immune dysregulation within holoendemic malarial regions. Because the conceptual frameworks converge observations from heterogeneous study designs, several of the proposed mechanistic relationships remain inferential. Despite this limitation, the framework illustrates several immune pathways that warrant further investigation as potential therapeutic or preventive targets. Future studies should validate the proposed mechanisms to guide the development of strategies aimed at reducing the burden of this aggressive cancer in high-risk populations.

## Data Availability

No datasets were generated or analysed during the current study.

## Abbreviations

ADCC: Antibody-dependent cellular cytotoxicity
AID: Activation-induced cytidine deaminase
APC: Antigen-presenting cells
BCR: B cell receptor
BACH2: BTB and CNC homolog 2
BL: Burkitt Lymphoma
BLGSP: Burkitt Lymphoma Genome Sequencing Project
CR2: Complement receptor type 2
CSR: Class-switch recombination
eBL: Endemic Burkitt Lymphoma
EBV: Epstein-Barr Virus
EFS: Event-free survival
GC: Germinal center
HLA: Human Leukocyte Antigen
IFN: Interferon
IgA: Immunoglobulin A
IgM: Immunoglobulin M
KIR: Killer-immunoglobulin receptor
MHC: Major histocompatibility complex
MMR: Mismatch repair pathway
NET: Neutrophil extracellular trap
NHL: Non-Hodgkin Lymphoma
NK: Natural Killer cell
OS: Overall survival
Pf: Plasmodium falciparum
PfEMP1: P. falciparum erythrocyte membrane protein 1
RBC: Red blood cell
ROS: Reactive oxygen species
sBL: Sporadic Burkitt Lymphoma
SHM: Somatic hypermutation
TGFBI: Transforming growth factor beta-induced
TLR9: Toll-like receptor 9
UNG: Uracil-DNA glycosylase
